# Ossifying fibrous epulis as an IgG4-related disease of the oral cavity: a case report and literature review

**DOI:** 10.1186/s12903-022-02041-4

**Published:** 2022-01-10

**Authors:** Yoshiko Ike, Takahiro Shimizu, Masaru Ogawa, Takahiro Yamaguchi, Keisuke Suzuki, Yu Takayama, Takaya Makiguchi, Masanori Iwashina, Satoshi Yokoo

**Affiliations:** 1 Department of Oral and Maxillofacial Surgery, and Plastic Surgery, Gunma University Graduate School of Medicine, 3-39-22, Showa-machi, Maebashi-City, Gunma 371-8511 Japan; 2 Clinical Department of Pathology, Gunma University Graduate School of Medicine, 3-39-22, Showa-machi, Maebashi-City, Gunma 371-8511 Japan

**Keywords:** Epulis, IgG4-RD of the oral cavity, IgG4(+) plasma cell, Histopathology, Serological test

## Abstract

**Background:**

Fibrous sclerosing tumours and hypertrophic lesions in IgG4-related disease (IgG4-RD) are formed in various organs throughout the body, but disease in the oral region is not included among individual organ manifestations. We report a case of ossifying fibrous epulis that developed from the gingiva, as an instance of IgG4-RD.

**Case presentation:**

A 60-year-old Japanese man visited the Department of Oral and Maxillofacial Surgery, Gunma University Hospital, with a chief complaint of swelling of the left mandibular gingiva. A 65 mm × 45 mm pedunculated tumour was observed. The bilateral submandibular lymph nodes were enlarged. The intraoperative pathological diagnosis of the enlarged cervical lymph nodes was inflammation. Based on this diagnosis, surgical excision was limited to the intraoral tumour, which was subsequently pathologically diagnosed as ossifying fibrous epulis. Histopathologically, the ossifying fibrous epulis exhibited increased levels of fibroblasts and collagen fibres, as well as infiltration by numerous plasma cells. The IgG4/IgG cell ratio was > 40%. Serologic analysis revealed hyper-IgG4-emia (> 135 mg/dL). The patient met the comprehensive clinical diagnosis criteria and the American College of Rheumatology and European League Against Rheumatism classification criteria for IgG4-RD. Based on these criteria, we diagnosed the ossifying fibrous epulis in our patient as an IgG4-related disease. A pathological diagnosis of IgG4-related lymphadenopathy was established for the cervical lymph nodes. Concomitant clinical findings were consistent with type II IgG4-related lymphadenopathy.

**Conclusions:**

A routine serological test may be needed in cases with marked fibrous changes (such as epulis) in the oral cavity and plasma cells, accompanied by tumour formation, to determine the possibility of individual-organ manifestations of IgG4-related disease.

## Background

IgG4-related disease (IgG4-RD) is a chronic inflammatory disorder characterised by high serum IgG4 levels and infiltration by IgG4(+) plasma cells. It was originally reported from Japan, although its exact epidemiology and pathology have not yet been fully elucidated. Despite the recent confirmation of an association of type 2 helper T (Th2) cells and regulatory T (Treg) cells with the pathophysiology of IgG4-RD, it is still ambiguous whether IgG4-RD is an autoimmune or allergic disease [1–3].

The comprehensive clinical diagnosis (CCD) criteria [1, 2] developed in Japan have been widely used to diagnose IgG4-RD. However, in some cases, typical pathological features cannot be detected, despite serum IgG4 and infiltrate IgG4(+) plasma cell levels meeting the diagnostic criteria, thereby making it extremely difficult for clinicians to reach a definitive diagnosis. Thus, in 2019, the American College of Rheumatology (ACR) and European League Against Rheumatism (EULAR) prepared a set of classification criteria aimed at the holistic identification of homogenous populations and not just single patients [4].

Fibrous sclerosing tumours and hypertrophic lesions are formed in organs throughout the body, but disease in the oral region is not included among the individual organ manifestations of IgG4-RD. Only six studies have associated oral sclerosing disease with IgG4-RD upon searching PubMed from 2000 until 2020 [5–10].

We encountered a patient with an oral sclerosing lesion and IgG4-related lymphadenopathy in the regional lymph nodes (submandibular lymph nodes) that diagnostically met both the CCD criteria and ACR/EULAR criteria.

## Case presentation

**Fig. 1:**
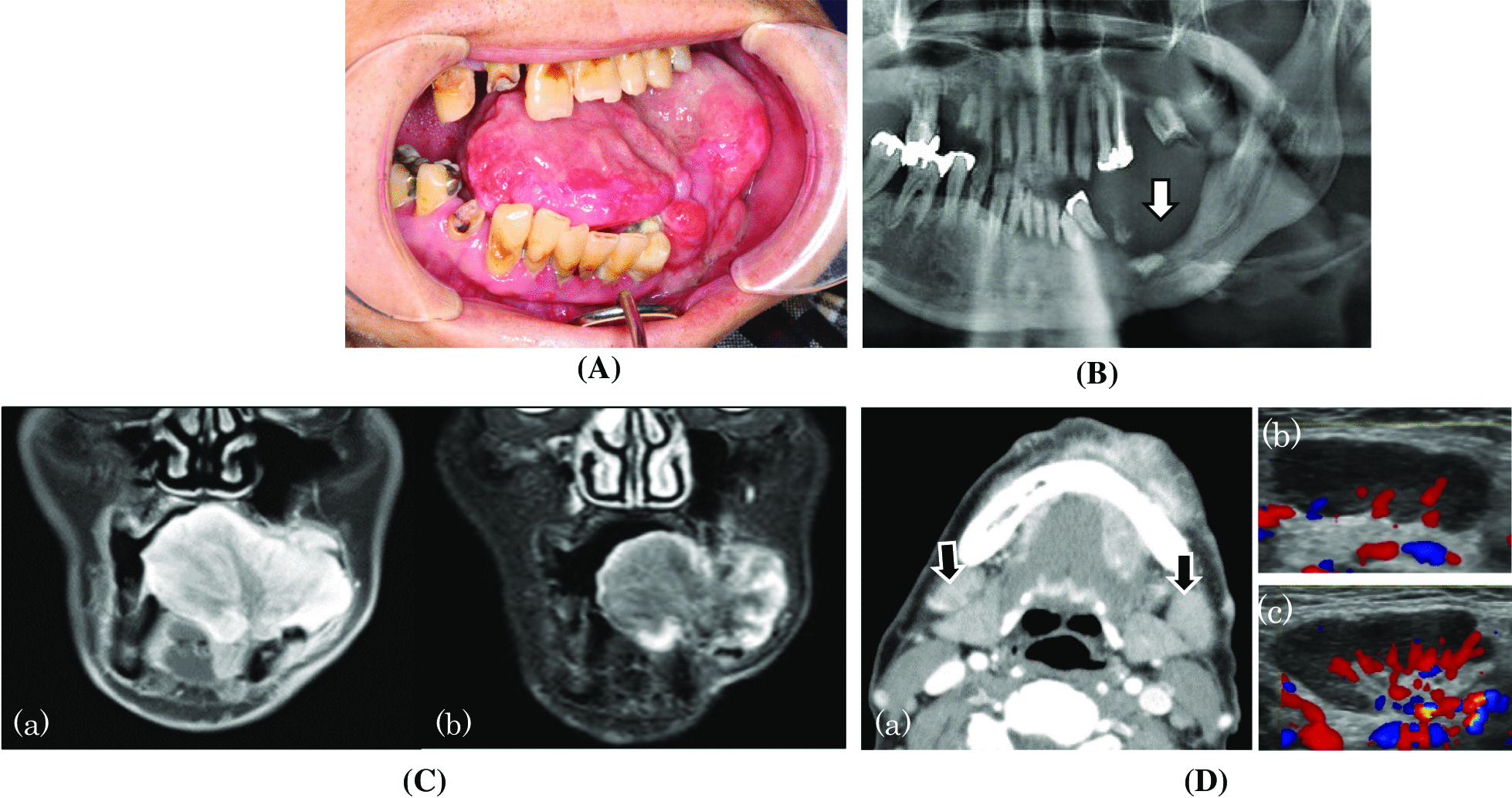
Patient status. **A** Oral findings at first examination: a pedunculated tumour (approximately 65 × 45 mm) is present in the left lower gingiva. The base is localised in the alveolar region, and the surface is granulomatous, elastic-hard with partial ulcerations, and haemorrhagic. The tongue is displaced rightward by the tumour (arrow). **B** Panoramic radiography revealed compressive bone resorption in the region corresponding to the tumour (arrow). **C** MRI images: (a) Contrast T1-Gd-MRI revealed significant contrast throughout the tumour, more pronounced along the margin; (b) T2-STIR-MRI revealed a high intensity at the margin and moderate intensity in the inner region. **D** Imaging of the submandibular lymph node: (a) in the contrast-enhanced CT image, contrast-enhanced swollen lymph nodes (arrows) are present in the bilateral submandibular regions. Both the right (b) and left (c) submandibular lymph nodes are soft, with smooth margins on cervical echo. The hilum of the lymph node is clear, but an increase in blood flow is noted in the hilum region. T1-Gd-MRI: T1-weighted gadolinium-enhanced magnetic resonance imaging, T2-STIR: T2-weighted short inversion recovery

A 60-year-old Japanese man with painful and enlarged intraoral tumour on the left side visited the Department of Oral and Maxillofacial Surgery, Gunma University Hospital. An unusually large pedunculated tumour of approximately 65 mm × 45 mm was localised in the left lower alveolar region. The tumour surface was granulomatous, elastic-hard, partially ulcerated, and haemorrhagic. The tongue was displaced rightward by the tumour (Fig. 1A). There was no hypoesthesia in the region innervated by the lower alveolar nerve. Oral hygiene was poor. Mobile elastic soft swollen lymph nodes, the size of the tip of the thumb, were palpated in the bilateral submandibular regions. The patient had a past medical history of oesophageal hiatal hernia and reflux oesophagitis, without asthma and allergy. He had no family history of particular relevance. His height was 160 cm and body weight was 53 kg. Difficulty with eating due to the tumour was noted, and the patient's nutritional status was poor.

Panoramic radiography and computed tomography (CT) scans both showed compressive bone resorption in the left mandible, but the cortical bone was retained (Fig. 1B). It was considered that the long-term persistent growth of the lesion had caused the compressive bone resorption. T1-weighted gadolinium-enhanced magnetic resonance imaging (T1-Gd-MRI) revealed significant contrast in the entire tumour, particularly in the tumour margin. A high marginal intensity was detected using short T1 inversion recovery (STIR) scanning (Fig. 1C). A positron emission tomography (PET)-CT scan showed high accumulation of fluorodeoxyglucose with a maximum standardised uptake value (SUVmax) of 5.7 in the region of the tumour. The CT scan of the cervical lymph nodes showed a swollen lymph node in both the left and right submandibular regions (approximately 25 mm and 20 mm respectively), with the swollen node on the right displaying high contrast. Cervical echo confirmed both the swollen submandibular lymph nodes to be flat, with smooth margins. The lymph node hila were clear, although a localised increase in blood flow was observed in the hilar region (Fig. 1D). PET-CT scan showed accumulation in the bilateral submandibular lymph nodes, with SUVmax values of 3.9 and 3.0 on the right and left sides, respectively.

**Fig. 2:**
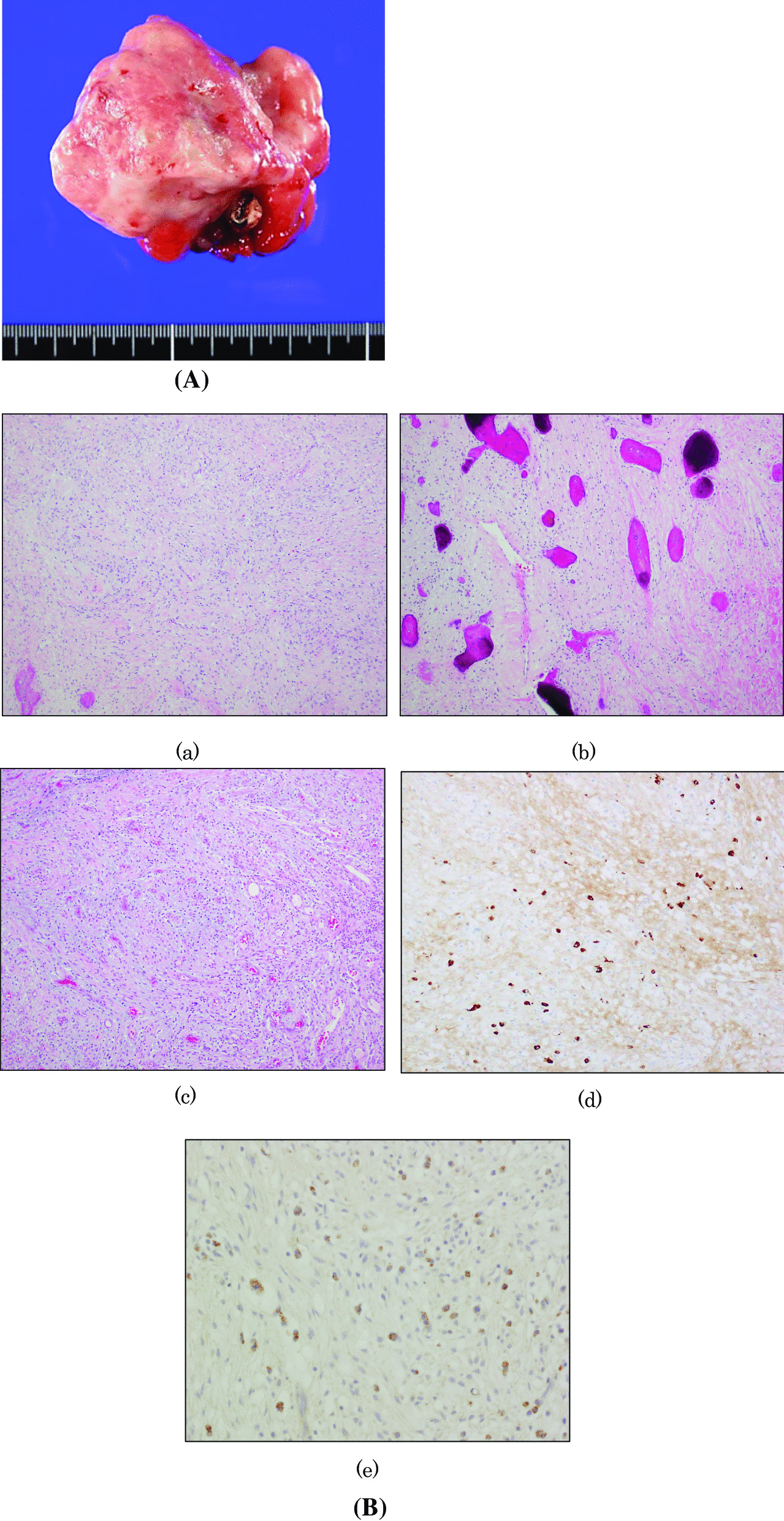
Findings for the excised oral lesions. **A** The excised oral tumour is an elastic hard pedunculated tumour. **B** Histopathological findings for the oral tumour: (a) there are fibroblasts and collagen fibres growing out of the subepithelial tissue, developing storiform fibrosis (× 100, HE staining). (b) Lamellar bone formation is observed among the outgrowth of fibrous tissue (× 100, HE staining). (c) There are reactive outgrowing blood vessels and infiltration by inflammatory cells (neutrophils and plasma cells) (× 100, HE staining). (d) The IgG4/IgG plasma cell ratio is approximately 62.3% (× 200, immunostaining). (e) TGF-β is expressed in many plasma cells (× 400, immunostaining)

The clinical diagnosis was a suspected malignant left lower gingival tumour with submandibular lymph node metastasis. The pathology of incisional biopsy indicated the possibility of a mesenchymal tumour, but no definite diagnosis could be made, because there was a strong inflammatory reaction. Thus, excision of the oral tumour (following the procedure for oral malignant tumours), and excisional biopsy of the left submandibular lymph node, were planned. Radical neck dissection was also planned in the event of detecting a malignancy in the submandibular lymph node during intraoperative rapid pathological diagnosis. Rapid pathological diagnosis of the submandibular lymph node during surgery revealed only inflammation but no sign of malignancy. The oral tumour was dissected by subperiosteal detachment with a 10 mm safety margin (Fig. 2A). The cortical bone was also scraped, and the region was covered with an absorbable polyglycolic acid sheet using a tissue adhesive.

The cut surface of the excised tumour was solid and whitish. Haematoxylin and eosin (HE) staining revealed the surface to be covered with stratified squamous epithelium and outgrowths of fibroblasts and collagen fibres, showing marked storiform fibrosis. No capsule was present, and lamellar bone formation was observed in the outgrowing fibres. Reactive hyperplasia of blood vessels and inflammatory cell infiltration, such as neutrophils and plasma cells, were also detected (Fig. 2Ba–c). Immunohistology revealed that numerous IgG(+) plasma cells and IgG4(+) plasma cells were stained with an IgG4/IgG ratio of approximately 62.3% (Fig. 2Bd). Forty-five IgG4(+) plasma cells were present per high-power field (HPF) at 400× magnification, with many expressing transforming growth factor beta (TGF-β) (Fig. 2Be). Generally, ossifying fibrous epulis exhibits reduced infiltration of inflammatory cells because of chronic inflammation. However, the present case showed marked infiltration of inflammatory cells with a high IgG/IgG4 plasma cell ratio and plasma cell numbers/HPF. Thus, the present case was diagnosed as ossifying fibrous epulis with a suspicion of IgG4-RD based on the other histopathological findings. Based on immunohistochemical findings, mesenchymal tumours, such as solitary fibrous tumour (antibody: bcl-2, CD34, STAT6) and myofibroma (antibody: SMA), were excluded.

**Fig. 3:**
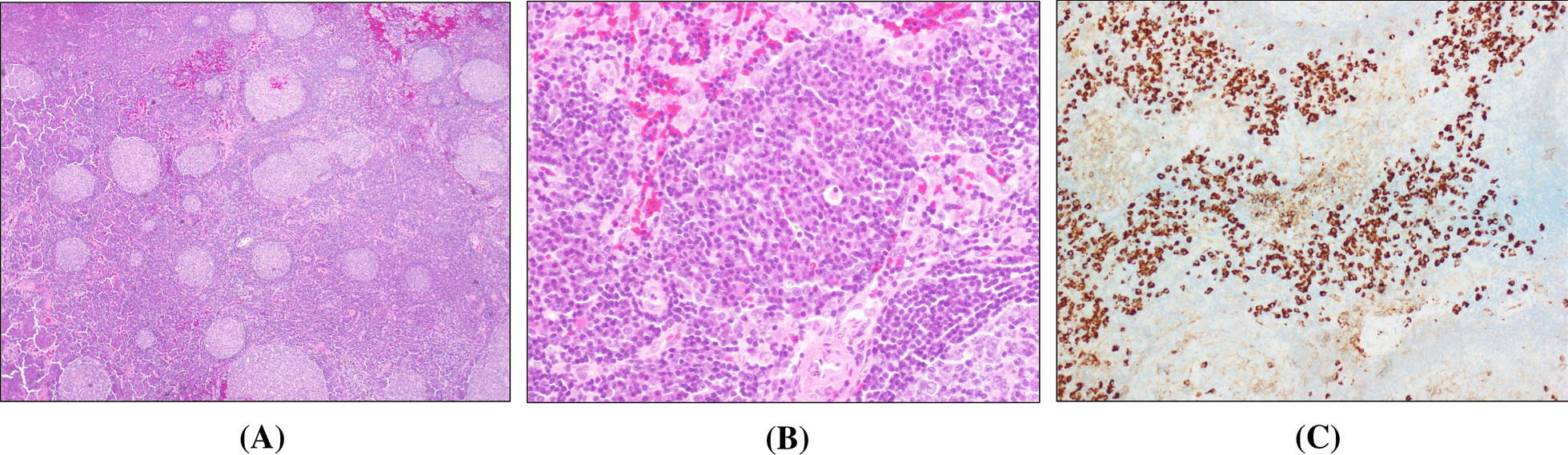
Histopathological findings for the submandibular lymph nodes. **A** Hyperplasia of the lymphoid follicles, which are variously sized accompanied by germinal centres (× 100, HE staining). **B** Infiltration by many inflammatory cells (mainly plasma cells) between follicles (× 400, HE staining). **C** The IgG4/IgG plasma cell ratio is approximately 87.3% (× 200, immunostaining)

The excised submandibular lymph node was soft, and the cut surface was solid and milky white. HE staining showed hyperplasia of the lymphoid follicles of irregular sizes accompanied by germinal centres. Blood vessels had grown between the lymphoid follicles and many plasma cells, and fewer eosinophils were mixed, showing a morphology similar to that of reactive lymphadenitis (Fig. 3A, B). Immunohistology detected numerous IgG4(+) plasma cells in the lymphoid follicles. The IgG4/IgG ratio was approximately 87.3%, and the IgG4(+) plasma cell count was 138/HPF (Fig. 3C). Blood tests showed a high serum IgG level of 2025 mg/dL, and a high serum IgG4 level of 312 mg/dL. A complete blood count was normal, c-reactive protein was mildly elevated to 1 0.83 mg/dL, and the albumin level was slightly low, at 3.1 g/dL. Serum IgA, IgM, and IL-6 levels were not elevated, while anti-SS-A and anti-SS-B antibodies were negative. IgG4-related lymphadenopathy cannot be easily differentiated from plasma cell-rich Castleman disease only by histopathological examination. In the present case, plasma cell-rich Castleman disease was excluded because of the absence of the conditions of hyperIL-6-emia, such as fever, persistently high CRP, microcytic anaemia, and thrombocytosis. Thus, the diagnosis of the submandibular lymph node was IgG4-related lymphadenopathy.

Based on this information, a whole-body examination was performed by the Department of Nephrology and Rheumatology, but no finding suggesting the presence of IgG4-RD was observed in any other organ. Since the lesion was localised and the residual right submandibular lymph node was not enlarged, the patient was subjected to course observation. Approximately seven years after surgery, the course has been favourable, without enlargement of the submandibular lymph nodes, or appearance of a new lesion, and without recurrence of ossifying fibrous epulis.

## Discussion and conclusions

The oral tumour (ossifying fibrous epulis) and submandibular lymph nodes in our patient met all three CCD criteria for IgG4-RD: (1) clinical examination revealing characteristic diffuse/localised swellings or masses in single or multiple organs; (2) haematological examination revealing elevated serum IgG4 levels (> 135 mg/dL); and (3) pathological examination revealing (a) marked lymphocyte and plasmacyte infiltration and fibrosis, and (b) infiltration by IgG4(+) plasma cells, with a ratio of IgG4(+)/IgG(+) plasma cells > 40%, and > 10 IgG4(+) plasma cells per HPF. Thus, both oral and cervical lesions in the present case were included in the definitive diagnosis.

Storiform fibrosis and obliterative phlebitis have been reported as other pathological characteristics of IgG4-RD, but their development often depends on the specific organ from which the disease develops [11]. We observed storiform fibrosis in the epulis of our patient, whereas obliterative phlebitis was not detected. However, disease in the oral region is neither included as an individual-organ manifestation of IgG4-RD [1–3], nor are its typical pathological characteristics well defined, since only six studies associate oral sclerosing disease with IgG4-RD to any substantial extent [4–9] (Table 1). Tokura [11] suggested that IgG4-related skin disease does not necessarily exhibit typical pathological characteristics, because the skin which consists of orthokeratosis on the epidermis, and which is in contact with the outside world, is influenced by numerous stresses, including bacteria, mechanical stress, and ultraviolet radiation. In IgG4-RD, sclerosing disease originating from the gingiva and hard palate may not have typical pathological features, because the epithelium in these regions shows parakeratosis, and also because they are exposed to oral bacteria and mechanical stress and other stressors. Oral sclerosing disease is not included among the ACR/EULAR inclusion criteria [4]. However, our case scored 33 for the inclusion criteria, thereby meeting the ACR/EULAR criteria. Wallace et al. [4], referring to data presented by Deshpande et al. [12], who introduced the ACR/EULAR criteria, mentioned that classic pathological findings were absent in 37% of the research patients. Based on these reports, and in conjunction our pathological findings, we diagnosed the ossifying fibrous epulis in the present case as an IgG4-RD.

**Table 1:** Reported cases of IgG4-RD in the oral cavity

Author (country)	Gender/Age	Localization	IgG4/1 HPF	IgG4/IgG (%)	Serum IgG4 level (mg/dL)	Other involved organs
Ono et al. 2012 (Japan) [5]	Male/65	Upper gingiva	135	**–**	171	Sclerosing lesion of the lung
Khurram et al. 2013 (UK) [6]	Female/45	Hard palate	Dense	65	352	Skin sclerosing lesion Cervical lymphadenopathy
Andrew et al. 2014 (Australia) [7]	Female/71	Minor salivary gland of the hard palate	280	80	3031	Sclerosing lesion of the lacrimal gland
Laco et al. 2015 (Czech Republic) [8]	Female/54	Floor of mouth	103	68	185	None
	Male/79	Lower gingiva	139	72	165	None
	Male/74	Upper gingiva	66	71	N/A	None
Gontarz et al. 2016 (Poland) [9]	Male/30	Upper gingiva	75	80	335	Cervical lymphadenopathy
Rampi et al. 2020 (Italy) [10]	Female/35	Hard palate	100	> 40	151	Sclerosing lesion of the pachymeninges, optic nerve, nasal septum
	Female/20	Hard palate	50	70	421	Cervical lymphadenopathy Sclerosing lesion of the parotid gland, oropharynx, pterygopalatine fossa
Present case	Male /60	Lower gingiva	45	62	312	Cervical lymphadenopathy

IgG4-RD: IgG4-related disease, N/A: not available

Strehl et al. [13] and Šteiner et al. [14] demonstrated the ubiquitous occurrence of a variably high number of IgG4(+) plasma cells under diverse non-specific inflammatory conditions. This indicates that high IgG4(+) plasma cell counts and high IgG4/IgG ratios do not reliably distinguish IgG4-associated systemic disease from non-specific conditions, and that IgG4 counts must be cautiously interpreted in the context of appropriate clinical and histopathological features. However, Strehl et al. [13] reported that the ratio of IgG4(+)/IgG(+) plasma cells in 12 patients with epulis plasmocellularis ranged from 18 to 77%, with a mean of 32%, and concluded from the low mean alone without further examination of cases with a high ratio. Serum IgG4 levels were also not measured in these patients. However, previous studies [5–10] establishing oral sclerosing disease as IgG4-RD had all emphasised the importance of serological testing in diagnosis, and had depended not only on the clinical and pathological findings based on the CCD criteria, but also on various serological findings for confirming their diagnosis. The present case had a high serum IgG4 level of 312 mg/dL. Therefore, to account for individual organ manifestations of IgG4-RD, a routine serological test may be needed in cases with marked fibrous changes, such as epulis in the oral cavity and plasma cells accompanied by tumour formation.

Immunohistopathological testing revealed that TGF-β was expressed in many plasma cells in our patient (Fig. 2Be). In IgG4-RD, infiltration by IgG4(+) plasma cells is initiated with an imbalance between type 1 helper T (Th1) cells and Th2 cells, with the Th2 cells gradually gaining dominance [15], followed by the induction of Treg cells to inhibit T2 cell dominance. However, the reasons behind the apparent dominance of the T2 cells and the presence of T cell-activating antigen are unclear. In the present case, we consider this to be the process whereby IgG4-RD developed. TGF-β (a Treg cytokine) activated the fibroblasts, thus inducing fibrosis, by causing B cell to differentiate into plasma cells via the actions of Th2 and Treg cells; this in turn caused class switching to IgG4-RD [16–23].

The disease-forming lesions in the lymph nodes observed in IgG4-RD are characteristic of a condition known as IgG4-related lymphadenopathy. IgG4-related lymphadenopathy is classified into five types based on clinical and pathological findings, and the type localised in the regional lymph nodes in IgG4-RD corresponds to type II. Since the epulis in the present cse was diagnosed as IgG4-RD of the oral cavity, and characteristic follicular hyperplasia and IgG4(+) plasma cell infiltration between follicles were detected, the present case was appropriately diagnosed with type II lymphadenopathy [24, 25].

In conclusion, in the present case, we diagnosed epulis that developed from the gingiva as IgG4-RD. A routine serological test may be required in cases with marked fibrous changes, such as epulis, in the oral cavity and plasma cells accompanied by tumour formation to evaluate possible individual organ manifestations of IgG4-RD.

## Data Availability

All data supporting the conclusions of this study are included within the article.

## Abbreviations

ACR: American College of Rheumatology
CCD: Comprehensive clinical diagnosis
EULAR: European League Against Rheumatology
HE: Haematoxylin and eosin
HPF: High-power field
IgG4-RD: IgG4-related disease
PET-CT: Positron emission tomography and computed tomography
SUVmax: Maximum standardised uptake value
T1-Gd MRI: T1-weighted gadolinium-enhanced magnetic resonance imaging
T2-STIR: T2-weighted short inversion recovery
TGF-β: Transforming growth factor beta
Th1: Type 1 helper T cell
Th2: Type 2 helper T cell
Treg: Regulatory T cell
